# Analysis of Exosomal MicroRNA Dynamics in Response to Rhinovirus Challenge in a Longitudinal Case-Control Study of Asthma

**DOI:** 10.3390/v14112444

**Published:** 2022-11-03

**Authors:** Wangfei Wang, Anirban Sinha, René Lutter, Jie Yang, Christian Ascoli, Peter J. Sterk, Nicole K. Nemsick, David L. Perkins, Patricia W. Finn

**Affiliations:** 1 Richard and Loan Hill Department of Biomedical Engineering, College of Engineering and Medicine, University of Illinois at Chicago, Chicago, IL 60607, USA; 2 Department of Pulmonary Medicine, Amsterdam University Medical Centers, Location Academic Medical Center, University of Amsterdam, 1105 AZ Amsterdam, The Netherlands; 3 Department of Experimental Immunology, Amsterdam University Medical Centers, Location Academic Medical Center, University of Amsterdam, 1105 AZ Amsterdam, The Netherlands; 4 Department of Mathematics, Statistics, and Computer Science, College of Liberal Arts and Sciences, University of Illinois at Chicago, Chicago, IL 60607, USA; 5 Division of Pulmonary, Critical Care, Sleep and Allergy, Department of Medicine, College of Medicine, University of Illinois at Chicago, Chicago, IL 60612, USA; 6 Department of Molecular and Cellular Biology, College of Liberal Arts and Sciences, University of Illinois at Chicago, Chicago, IL 60607, USA; 7 Division of Nephrology, Department of Medicine, College of Medicine, University of Illinois at Chicago, Chicago, IL 60612, USA

**Keywords:** microRNA, exosome, rhinovirus, asthma, dynamics, longitudinal, case-control study, pulmonary

## Abstract

Asthma symptoms are often exacerbated by the common-cold-causing rhinovirus (RV). In this study, we characterized the temporal behavior of circulating exosomal microRNAs (ExoMiRNAs) in a longitudinal bi-phasic case-control study of mild asthmatics (*n* = 12) and matched non-atopic healthy controls (*n* = 12) inoculated with rhinovirus. We aimed to define clinical and immunologic characteristics associated with differentially expressed (DE) miRNAs. In total, 26 DE ExoMiRNAs, including hsa-let-7f-5p, hsa-let-7a-5p, hsa-miR-122-5p, hsa-miR-101-3p, and hsa-miR-126-3p, were identified between asthmatic and healthy subjects after inoculation with RV. Time series clustering identified a unique Cluster of Upregulated DE ExoMiRNAs with augmenting mean expression and a distinct Cluster of Downregulated DE ExoMiRNAs with mean expression decline in asthmatic subjects upon RV challenge. Notably, the Upregulated Cluster correlated with Th1 and interferon-induced cytokines/chemokines (IFN-γ and IFN-γ-inducible protein-10) and interleukin-10 (IL-10). Conversely, the Downregulated Cluster correlated with IL-13, a Th2 cytokine, pulmonary function measurements (FVC%, FEV1%, and PEF%), and inflammatory biomarkers (FeNO, eosinophil%, and neutrophil%). Key ExoMiRNA–target gene and anti-viral defense mechanisms of the Upregulated and Downregulated Clusters were identified by network and gene enrichment analyses. Our findings provide insight into the regulatory role of ExoMiRNAs in RV-induced asthma.

## 1. Introduction

Asthma is a chronic inflammatory pulmonary disease with allergic asthma being the most common form [1]. The dynamic interaction between the endogenous and environmental factors can result in episodic asthma symptoms called loss of control or exacerbations [2]. These are acute, progressive worsening of symptoms that can be life-threatening if not treated promptly. Respiratory viral infections, such as the prevalent common-cold-causing rhinovirus, are risk factors for asthma exacerbations and unique immune responses may be fundamental in the pathogenesis of RV-induced exacerbations of asthma [3].

RV infections induce abnormal cytokine responses in asthma, including impaired Th1 cytokine production [4,5,6]. As such, studies have shown that immune response phenotypes characterized by deficient IFN-γ and IL-6 are associated with worse clinical outcomes and higher risk of asthma exacerbations [5,7]. Furthermore, responses of biomarkers for airway inflammation and asthma phenotypes such as fractional exhaled nitric oxide (FeNO) and the eosinophil and neutrophil percentages differ in asthmatics compared to their healthy counterparts [8,9,10,11].

Recently, exosomes, small membrane-bound vesicles (∼40 to 160 nm), have emerged as important mediators of intercellular communication given their potential to deliver various molecules between cells, including microRNAs (miRNAs) [12]. Exosomal miRNAs (ExoMiRNAs) are small non-coding RNAs (∼18 to 25 nucleotides in length) that may participate in post-transcriptional gene regulation upon delivery to target cells and as a result impact the local and systemic inflammatory milieu [13,14]. Consequently, ExoMiRNAs have been demonstrated to play important roles in immune regulation, inflammation, and anti-viral immunity in asthma [15,16,17]. Despite the implications of ExoMiRNAs in asthma, little is known about how miRNAs respond to common triggers of asthma exacerbations such as respiratory viral infections (e.g., RV) and how miRNAs are linked to cytokine responses and inflammatory biomarkers over time.

Therefore, to investigate the host ExoMiRNA responses to RV infection, we designed a longitudinal bi-phasic case-control study [18,19]. We challenged asthmatic subjects and healthy controls with RV in vivo, and analyzed ExoMiRNAs expression, cytokine production, and inflammatory biomarkers. We hypothesize that ExoMiRNAs are differentially expressed (DE; includes upregulated and downregulated) and that time series clustering analysis of the miRNAs may reveal clusters that are associated with features of asthma exacerbations. Moreover, miRNA clusters may identify different key target genes and pathways implicated in anti-viral and defense responses to RV infection.

## 2. Materials and Methods

### 2.1. Human Subjects and Sample Collection

The study was approved by the medical ethical committee from the Amsterdam University Medical Center (AUMC), location Academic Medical Center (University of Amsterdam), and has been registered at the Netherlands Trial Register (NTR5426/NL5317). Twelve subjects with mild allergic asthma and twelve well-matched non-atopic healthy controls were recruited into an observational, longitudinal bi-phasic case-control study [18]. The study involved the assessment of subjects at baseline and thereafter thrice weekly for 2 months followed by stimulation with RV16 (pre- and post-viral challenge phases) and similar follow-up for the subsequent month. Serum samples ($\bar{x}=8$ serum samples per patient before the rhinovirus challenge and $\bar{x}=4$ serum samples per patient after rhinovirus challenge) with variable time intervals were collected. Baseline characteristics of healthy and asthmatic subjects and the detailed inclusion and exclusion criteria were listed in Sinha et al. [18].

Other biological samples and clinical and inflammatory markers were also sampled longitudinally. Ten cytokines/chemokines from nasal lavage were measured: interferon-gamma (IFN-γ), interleukin (IL)-1β, IL-10, IL-13, IL-17A, IL-33, IL-6, IL-8, IFN-γ-inducible protein-10 (IP-10), and tumor necrosis factor alpha (TNF-α) [19]. Additionally, fractional exhaled nitric oxide (FeNO) and eosinophil and neutrophil percentages in nasal lavage were measured [18]. Pulmonary function parameters measured include FVC, FEV${}_{1}$, and PEF [18]. For the detailed protocol of clinical data collection and clinical traits measurement, refer to Sinha et al. [18].

### 2.2. Exosomal miRNA Extraction, Library Preparation, and Sequencing

Exosomal RNAs (exoRNAs) were extracted using ExoQuick Exosome Isolation and RNA Purification Kit (System Biosciences, Palo Alto, CA, USA) from 500 μL serum following the manufacturer’s protocol. Exosomes were precipitated from serum, and then lysed to release and purify the RNA. For exosome characterization, please see Supplementary Materials. A small RNA library was prepared using NEBNext Multiplex Small RNA Library Prep Set (New England Biolabs, Ipswich, MA, USA) according to the protocol with 15 PCR amplification cycles. The quality control check was performed using Bioanalyzer High Sensitivity DNA Kit (Agilent, Santa Clara, CA, USA). Samples were pooled in equal amounts (pg) and small RNAs were then selected with peak size ∼145 bp using 3% agarose gel cassettes on the Pippin Prep instrument (Sage Science, Beverly, MA, USA). Single-end reads were generated from Illumina Miseq using MiSeq Reagent Kits v2 (Illumina, San Diego, CA, USA).

### 2.3. miRNA-seq Annotation and Differential Expression (DE) Analysis

Adaptors were removed with Cutadapt [20], and then sequences were mapped to the whole human genome GRCh38 with Bowtie [21]. MiRNA counts were generated by using the Python package Htseq-count [22] by searching against the miRNA feature file from miRbase database [23].

The low-count miRNAs were filtered and normalized in DESeq2 [24]. The normalized expression level of each miRNA was fitted using smoothing splines in R package “gss” [25] using individual effect as a random effect. The likelihood ratio test was performed using the maximum likelihood estimations from the reduced model and the full model to calculate the *p*-value of each miRNA. For model variables, “Group” (healthy or asthmatic subjects), “Challenge” (before or after RV challenge), “Group × Challenge” (regarded as a new variable instead of an interaction of main effects), and “Batch” (sequencing batch) were treated as factors. For example, to test whether the miRNA was differentially expressed before and after the RV challenge in healthy subjects, we tested the full model: miRNA∼Time+Group+Challenge+Group×Challenge+Run|Individual, and the reduced model: miRNA∼Time+Group+Group×Challenge+Run|Individual, where Individual was treated as a random effect. We performed the following comparisons: whether the miRNA was differentially expressed before and after the RV challenge in healthy subjects and asthmatic subjects, respectively, and whether the miRNA was differentially expressed between asthmatic and healthy subjects at baseline and after the RV challenge, respectively. To adjust for multiple comparisons, we used the Bonferroni correction by setting the critical value as 0.05/n, where n is the total number of tests [26].

### 2.4. Time Series Clustering of DE miRNAs

To study the dynamics of miRNA expression, time series clustering of DE miRNAs between asthmatic and healthy subjects after the RV challenge was performed using the R package Mfuzz [27]. Mfuzz is a soft clustering method based on fuzzy c-means. The log2 fold change of miRNAs between asthmatic and healthy subjects after the RV challenge was used to generate clusters. The miRNAs that have membership values over 0.55 were assigned to one cluster. To analyze and visualize the mean trend of the miRNA clusters, we performed functional principal components analysis using PACE (principal components analysis through conditional expectation [28]; fdapace in R [29,30]).

### 2.5. miRNA–Traits Correlation Analysis

Canonical correlation analysis was used to assess the correlations between temporal sequences of clinical traits and DE miRNAs. Canonical correlation analysis finds pairs of linear combinations of miRNAs and linear combinations of clinical traits that are highly correlated. Wilks’ lambda was used to test whether the correlations between miRNAs and clinical traits were significant. Only the first canonical variate pair of miRNAs and clinical traits was considered. The sets of clinical traits considered in canonical correlation analysis were cytokines, inflammatory biomarkers (eosinophils%, neutrophils%, FeNO), and pulmonary function measurements (FVC% (predicted), FEV${}_{1}$% (predicted), and PEF% (predicted)). Canonical correlation analysis was performed in SAS 9.4.

### 2.6. MiRNA Gene Target Prediction and Gene Network Construction

MiRNA gene targets were first predicted using TargetScan 7.1 [31]. Two miRNAs, hsa-miR-126-3p and hsa-miR-101-3p, were not present in TargetScan, and Tarbase v8 [32] was used for these two miRNAs. For target genes and gene ontology (GO) pathways, to increase the likelihood of identifying miRNA–mRNA interactions relevant to RV-induced asthma, we utilized an independent cohort of mild to moderate asthmatics and included only interactions with DE mRNAs [33]. In that study, peripheral blood cells were collected from 20 patients, before RV16 (identical dose as used in the current study) challenge and 6 days after the challenge, and performed RNA isolation and sequencing analysis as described in Ravi et al. [33]. A total of 674 differential expressed genes (DEGs) for the comparison before the RV16 challenge and 6 days after the challenge were identified (Table S5 in Ravi et al. [33]). We created an miRNA–target gene network in Cytoscape [34]. The target genes were analyzed for enriched GO terms in DAVID Bioinformatics Resources [35,36].

### 2.7. Statistical Analysis

Mixed-effects regression models were used to fit each cytokine, inflammatory biomarker, and pulmonary function measurement. The expression levels of these clinical traits before the rhinovirus challenge were averaged to create the baseline level. For cytokine response, subject group, linear and quadratic terms for time (time and time × time), and interaction of the subject group and the linear and quadratic terms for time (group-by-time and group-by-time × time interaction) were considered as fixed effects in the models. The quadratic term for time was removed when the *p*-value was greater than 0.05 in backward elimination. For inflammatory biomarkers and pulmonary function measurements, only the subject group, linear term for time, and group-by-time interaction were considered due to the lack of quadratic time trend. Time was modeled as a continuous variable. Random intercepts for each subject were included in all models. Random slopes for each subject were kept in the final model only when the model generated the smallest Akaike information criterion (AIC). All clinical measurements were log-transformed to promote the normality of the residuals of the error in mixed-effects regression models. Models with combinations of different random effects (random intercepts with and without random slopes) and different error variance–covariance structures (within-person correlations) were constructed. The candidate model was selected based on lower AIC or using likelihood ratio tests between nested models. The final model was selected using a likelihood ratio test between the candidate model and the unstructured saturated model. Mixed-effects regression analysis was performed in SAS 9.4.

## 3. Results

### 3.1. On Average the RV-Induced Cohort Showed No Significant Pulmonary Function Decline after RV Infection

Twelve subjects with mild allergic asthma and twelve well-matched healthy controls were inoculated with RV16. The baseline demographic characteristics are shown in Table 1 and no significant differences were found between the healthy and asthmatic subjects. Mixed-effects models were used to investigate whether RV-induced pulmonary function declined. At baseline, we did not observe a significant difference in pulmonary function measurements (Figure S1, Table 2, $\beta_{\mathrm{Group}}$, *p*-value > 0.05). Following the RV challenge, subjects with mild/well-controlled asthma did not show pulmonary function decline compared to their healthy counterparts (Table 2, $\beta_{\mathrm{Time}\times \mathrm{Group}}$, *p*-value > 0.05; Figure S1D). Among the three pulmonary function measurements, only FVC% was attenuated by the RV challenge (Table 2, $\beta_{\mathrm{Time}}$, *p*-value = 0.0146), while FEV${}_{1}$% and PEF% were not altered.

### 3.2. RV Elicited a Neutrophilic Response in Asthmatic Subjects When Sampled Longitudinally

**Figure 1:**
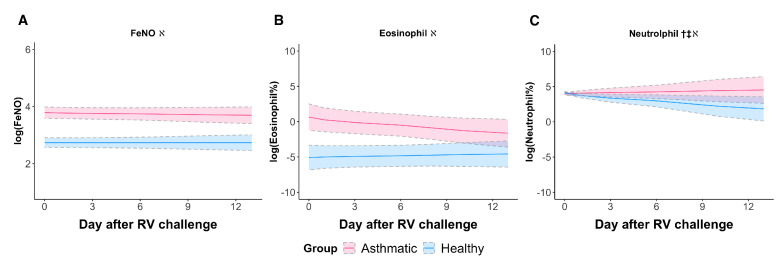
Inflammatory biomarkers in response to the RV challenge in asthmatic (magenta) and healthy subjects (blue). Mixed-effects models for log-transformed levels of (**A**) FeNO, (**B**) eosinophil percentage, and (**C**) neutrophil percentage in asthmatic (magenta) and healthy subjects (blue) showing significantly higher FeNO and eosinophil and neutrophil percentages in asthmatics at baseline and an increasing level of neutrophil percentage in asthmatics post-RV challenge. Day 0 represents the averaged levels at baseline. The solid lines represent the fitted trajectories of response. Upper and lower 95% confidence intervals are indicated by dashed lines. The results of mixed-effects regression models are shown in Table 2. The symbol † represents the measuring response in asthmatic and healthy subjects was significantly different after the RV challenge (Time × Group variable in Table 2), ‡ represents the RV challenge induced the measuring response significantly in healthy subjects (Time variable in Table 2), and *ℵ* represents asthmatic and healthy subjects were significantly different at baseline (Group variable in Table 2).

To investigate inflammation in the upper airway and lower airway compartments, we analyzed the changes in inflammatory biomarkers (inflammatory cell content, including eosinophil and neutrophil in nasal lavage fluid, and FeNO) after the RV challenge. Neutrophil percentage showed a significant increase in asthmatic subjects compared to healthy subjects following the RV challenge (Figure 1C, $\beta_{\mathrm{Time}\times \mathrm{Group}}$, *p*-value = 0.0435). However, FeNO and eosinophil percentages in nasal lavage did not differ among asthmatic and healthy subjects following the RV challenge. At baseline, FeNO and nasal lavage eosinophil percentage in the asthmatic subjects were significantly higher than those in healthy subjects (Table 2, $\beta_{\mathrm{Group}}$, *p*-value < 0.05, Figure 1A). Interestingly, the RV challenge failed to perturb FeNO and eosinophil percentage levels (Figure 1A,B, $\beta_{\mathrm{Time}}$, *p*-value > 0.05).

### 3.3. Rhinovirus Suppressed IFN-γ and IL-6 Responses and Impaired the IL-17A Response in Asthmatics

**Figure 2:**
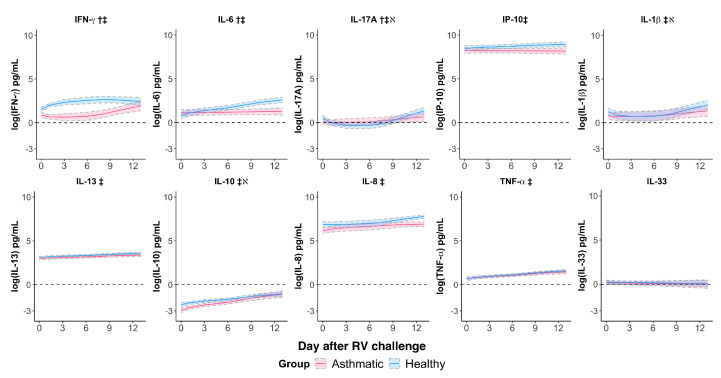
Mixed-effects models for log-transformed cytokine levels in asthmatic (magenta) and healthy subjects (blue) after the RV challenge. IFN-γ, IL-17A, and IL-6 (†) expressions in asthmatic subjects were significantly different from those in healthy subjects in response to RV challenge. All cytokines expect for IL-33 were induced by the RV challenge (‡). IL-17A, IL-1β, and IL-10 expression levels were significantly different at baseline (*ℵ*). Day 0 represents the averaged cytokine levels at the baseline. The solid lines represent the fitted trajectories of cytokine expressions in the asthmatic subjects (magenta) and healthy subjects (blue). Upper and lower 95% confidence intervals are indicated by dashed lines. The estimates and significance of the coefficients of mixed-effects regression models are shown in Table 2.

We assessed the patterns of cytokine responses from the first two weeks (∼13 days) after the RV challenge using mixed-effects models (Figure 2, Table 2). Four cytokines, IFN-γ, IL-17A, IL-1β, and IL-8, had a quadratic trend after the RV challenge, while the other cytokines, IP-10, IL-13, IL-10, IL-6, TNF-α, and IL-33, had a linear trend. At baseline, IL-17A, IL-1β, and IL-10 expression levels were significantly lower in asthmatics (Figure 2, Table 2, $\beta_{\mathrm{Group}}$, *p*-value < 0.05). The RV challenge did not induce changes in expression of the T-helper cell type 2 (Th2)-upstream cytokine IL-33, indicating RV infection did not promote type 2 inflammation via IL-33. However, the RV challenge induced a wide range of immune responses in asthmatic and healthy subjects suggested by the induction of significant changes in the other nine cytokines.

We then examined whether RV challenge induced significant changes in expressions of cytokines between asthmatic and healthy subjects (Table 2, $\beta_{\mathrm{Time}\times \mathrm{Group}}$). The expression levels of three cytokines, IFN-γ, IL-17A, and IL-6, in asthmatic subjects significantly differed from those in healthy subjects after the virus challenge. RV challenge induced a robust IFN-γ and IL-6 response in healthy subjects, while their response was dampened in asthmatic subjects (Figure 2). Interestingly, after the RV challenge, IL-17A was downregulated in healthy subjects, but not in the asthmatics. Following the RV challenge, IL-13, IL-10, IL-8, and TNF-α had a common increasing trend among asthmatic and healthy subjects. RV challenge induced an increased level of IP-10 in healthy subjects, but its effect on asthmatic subjects was marginal. The IP-10 level between asthmatic and healthy subjects was not significant (*p*-value = 0.0744). The trends of IL-1β differed between two subject groups following the RV challenge, but the difference was borderline significant (*p*-value = 0.0562). Taken together, our findings showed that RV infection induced aberrant host immune responses mediated by IFN-γ, IL-17A, and IL-6 in asthma.

### 3.4. Identification of Differentially Expressed Systemic Exosomal miRNAs (ExoMiRNAs)

Nanoparticle tracking analysis of our laboratory controls showed the typical size of the exosomes (30–150 nm; Figure S2A,B). Western blot also demonstrated the expression of CD9, CD81, and HSP70 (data not shown).

**Figure 3:**
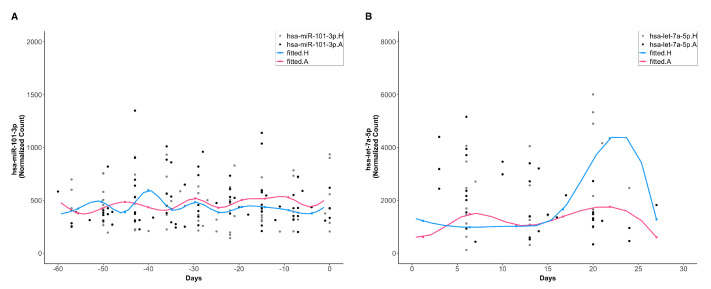
Representative trends of differentially expressed miRNAs among cohort groups. (**A**) No differential miRNA expression was found between asthmatic and healthy subjects at baseline (between-unchallenged). The figure shows hsa-miR-101-3p as an example. (**B**) Differential miRNA expression between asthmatic and healthy subjects at the virus-challenged phase (between-challenged). The blue and red lines are the fitted smoothing splines for healthy and asthmatic subjects, respectively. The gray and black dots are the observed normalized hsa-let-7a-5p counts from 12 healthy and 12 asthmatic subjects, respectively. The X-axis (days) represents the time interval between sample collection and virus challenge, with non-positive days indicating the baseline, and positive days indicating the virus challenged phase.

Next, we compared the ExoMiRNA expression levels between asthmatic and healthy subjects at two temporal phases using likelihood ratio tests. Strikingly, at baseline, no miRNA was differentially expressed (DE) between asthmatic and healthy subjects (between asthmatic and healthy subjects comparison at baseline (between-unchallenged); Figure 3A). After the RV challenge, a total of 26 miRNAs including hsa-let-7f-5p, hsa-let-7a-5p, hsa-miR-122-5p, hsa-miR-101-3p, and hsa-miR-126-3p were differentially expressed between asthmatic and healthy subjects (between asthmatic and healthy subjects comparison after RV challenge (between-challenged); Figure 3B; Table S1 contains the full list of these DE miRNAs).

We also examined whether RV perturbed the circulating ExoMiRNA expression within each of the subject groups. In healthy subjects, we identified a total of 49 miRNAs, including hsa-let-7a-5p, hsa-miR-451a, hsa-miR-151a-3p, hsa-let-7f-5p, hsa-let-7b-5p, hsa-miR-148a-3p, hsa-miR-126-3p, and hsa-miR-122-5p, whose expression was significantly different between pre- and post-RV challenged phases (pre- versus post-RV challenge within healthy subjects (within-healthy); Figure S3A). In asthmatic subjects, 64 miRNAs including hsa-let-7a-5p, hsa-miR-148a-3p, hsa-let-7f-5p, hsa-miR-26a-5p, hsa-miR-451a, hsa-miR-151a-3p, hsa-miR-21-5p, and hsa-miR-92a-3p were differentially expressed between pre- and post-RV challenged phase (pre- versus post-RV challenge within asthmatic subjects (within-asthmatic); Figure S3B).

Interestingly, 45 miRNAs were found to be in both the within-healthy and the within-asthmatic groups (Figure S3C). Intriguingly, all the 26 miRNAs in the between-challenged group were also in the within-asthmatic group (Figure S3C). Not only were these miRNA expression levels perturbed by RV in asthmatics, but they also differentiated from those in healthy subjects following the RV challenge, indicating their roles in RV-mediated infections. Therefore, we focused on the miRNAs in the between-challenged group and will henceforth refer to them as DE miRNAs.

### 3.5. Temporal Clustering Analysis of DE miRNAs Revealed Two Clusters of miRNAs with Different Dynamics and Correlations with Clinical Traits

**Figure 4:**
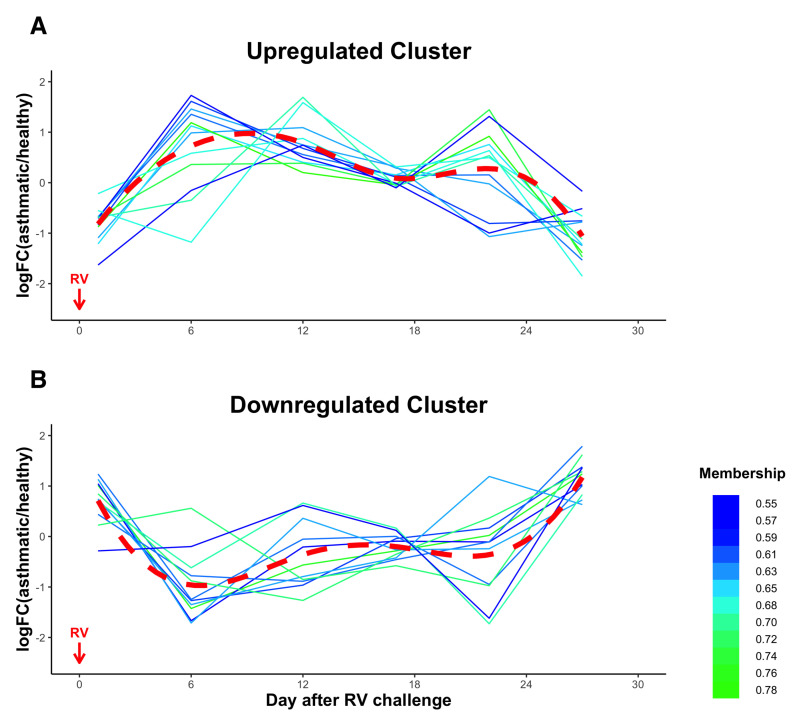
Differentially expressed miRNAs dynamics in response to RV. (**A**) The Upregulated Cluster has an upward mean trend up to 9 days after the RV challenge. (**B**) The Downregulated Cluster has a downward mean trend in expression up to 6 days after the RV challenge. Each line represents the log2 fold change of a miRNA in asthmatic subjects relative to healthy subjects. The X-axis (days) represents the time interval between sample collection and virus challenge. The color of the lines represents membership values. The larger the value is, the stronger the membership is for a given miRNA. The dotted red line represents the mean trend of the log2 fold change.

To guide the analysis of the functional dynamics of the 26 DE miRNAs, we performed fuzzy c-means clustering analysis on the longitudinal miRNA expression levels. We identified two clusters (12 miRNAs in the Upregulated Cluster and 11 miRNAs in the Downregulated Cluster) based on the log2 fold change of the miRNA expression levels between asthmatic and healthy subjects (Figure 4, Table 3). Three DE miRNAs, hsa-miR-223-5p, hsa-miR-425-3p, and hsa-miR-451a, were not clustered. In the Upregulated Cluster, the mean miRNA expression level in asthmatics was initially downregulated on day 1 post RV challenge compared to their healthy counterparts, and then had an increasing trend up to day 9 following RV challenge. Instead, the Downregulated Cluster miRNAs had an upregulated mean expression on day 1 post RV challenge and then had a decreasing trend up to day 6 following RV challenge in asthmatic subjects indicating two distinct classes of miRNA dynamics.

**Figure 5:**
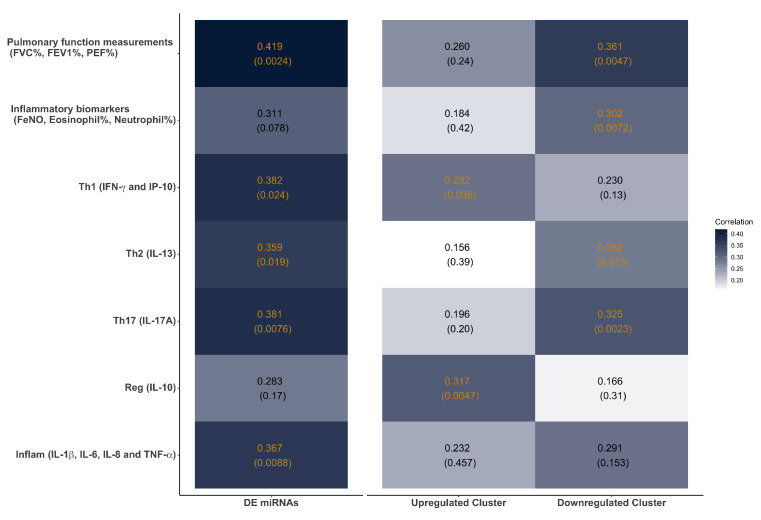
Canonical correlation analysis of miRNA and traits. The figure shows the adjusted canonical correlations between the miRNA group (DE miRNAs, Upregulated Cluster, and Downregulated Cluster) and clinical traits. The *p*-values (shown in the parenthesis) were tested from the Wilks’ lambda statistic. *p*-value that is smaller than 0.05 was considered significant and labeled in orange.

Identifying miRNA–clinical traits correlations is crucial to explore the mechanistic functions of the DE miRNAs related to clinical traits. Here, we employed canonical correlation analysis to assess the relationship between the temporal expression of DE miRNAs and the temporal fluctuations of pulmonary function, inflammatory biomarkers, and cytokines (Figure 5). To facilitate interpretation of the cytokine functions, we categorized the nine significant cytokines into five groups (IL-33 was removed because of the lack of response to RV challenge): (1) Th1 and interferon-induced cytokine/chemokine (Th1; IFN-γ, IP-10); (2) Th2 cytokine (Th2; IL-13); (3) Th17 cytokine (Th17; IL-17A); (4) regulatory cytokine (Reg; IL-10); and (5) proinflammatory cytokines (Inflam; IL-1β, IL-6, IL-8, and TNF-α). Interestingly, the DE miRNAs were significantly correlated with pulmonary function measurements, Th1, Th2, Th17, and Inflam, but not inflammatory biomarkers and the Reg cytokine group. Furthermore, analysis of correlations between different miRNA clusters and clinical traits revealed that two miRNA clusters were correlated with distinct sets of clinical traits. Upregulated Cluster miRNAs were significantly correlated with Th1 and Reg cytokine groups, while Downregulated Cluster miRNAs were significantly correlated with pulmonary function measurements, inflammatory biomarkers, and Th2 and Th17 cytokine groups. Downregulated Cluster miRNAs were correlated with all three pulmonary function measurements, FVC%, FEV${}_{1}$%, and PEF% (Table S4). Among the three inflammatory biomarkers, FeNO was driving the significant correlation with Downregulated Cluster miRNAs (correlation = 0.384), while eosinophil and neutrophil did not contribute to the significance (Table S4).

### 3.6. Functional Analysis of Gene Targets of DE miRNAs and Network Analysis Revealed Enriched Anti-Viral Defense Mechanisms

MiRNA hsa-miR-375-3p was not present in TargetScan or Tarbase and was therefore excluded from network analysis. Of the DE miRNAs, a total of 362 unique gene targets were identified (Table S5). The network of Upregulated Cluster miRNAs is comprised of 10 miRNAs and 208 unique target genes (Table S6), and the network of Downregulated Cluster miRNAs is comprised of 9 miRNAs and 226 unique target genes (Table S7). The gene hubs targeted by the greatest number of miRNAs were RORA and AGO1 in the Upregulated Cluster (Figure S4A), and FKBP5 and DICER1 in the Downregulated Cluster (Figure S4B). Increasing levels of RORA (retinoic acid receptor-related orphan receptor alpha (RORα)) and decreasing levels of AGO1, DICER1, and FKBP5 were observed after the RV challenge. RORA is a key transcription factor involved in regulating Th17 and ILC2 cells development and may mediate type 2 inflammation in asthma [37,38]. Lower expression of FKBP5 (FK506-binding protein 51 (FKBP51)) was found to reflect eosinophilic inflammation [39]. AGO1 and DICER1 are part of miRNA machinery and Dicer may exhibit miRNA-independent anti-viral function [40]. The Upregulated Cluster has a dominant group of gene targets by hsa-miR-221-5p and hsa-miR-126-3p, while the Downregulated Cluster has the most genes targeted by hsa-miR-4433b-3p and hsa-miR-101-3p.

**Figure 6:**
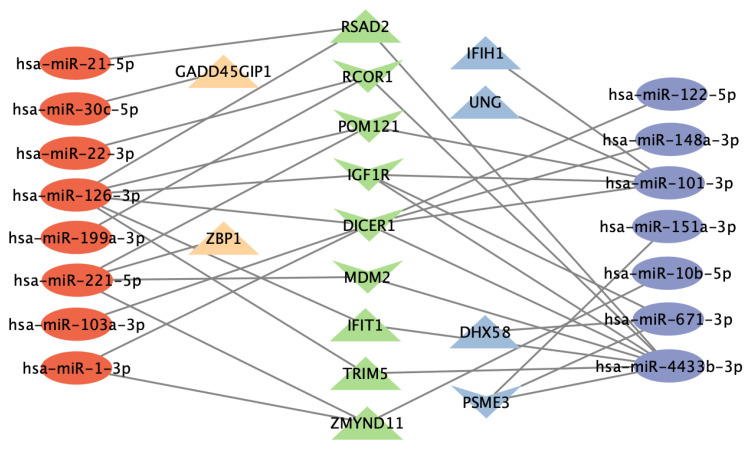
The network of DE miRNAs involved in enriched GO term “viral process”. Gene enrichment analysis was performed on gene targets of each DE miRNA. The full miRNA-gene regulatory networks are listed in Figure S4. Only the miRNAs and target genes involved in the GO term “viral process” are listed here. The labels are red circle (miRNAs in the Upregulated Cluster), purple circle (miRNA in the Downregulated Cluster), orange (genes targeted by Upregulated Cluster miRNAs only), blue (genes targeted by Downregulated Cluster miRNAs only), green (genes targeted by both the Upregulated Cluster and the Downregulated Cluster), triangle (genes upregulated at day 6 after the RV challenge compared to baseline), and “V” shape (genes downregulated at day 6 after RV challenge compared to baseline).

Gene functional annotation analysis of the gene targets of DE miRNAs revealed important enriched gene ontology (GO) terms related to anti-viral responses. The top five GO terms were “negative regulation of viral genome replication”, “response to virus”, “defense response to virus”, and “type I interferon signaling pathway” (Table S8). Functional analysis of the gene targets of the Upregulated Cluster and Downregulated Cluster miRNAs revealed common GO terms such as “viral process”, “defense response to virus”, and “type I interferon signaling pathway” (Table 4, Tables S9 and S10). “Viral process” was enriched by Upregulated Cluster miRNAs via GADD45GIP1 and ZBP, and by Downregulated Cluster miRNAs via IFIH1, UNG, DHX58, and PSME3. These two clusters of miRNAs also regulate a list of common genes including RSAD2, RCOR1, POM121, IGF1R, DICER1, MDM2, IFIT1, TRIM5, and ZMYND11 (Figure 6). The Upregulated Cluster and the Downregulated Cluster target many common genes and enriched GO terms that are relevant to anti-viral defense mechanisms.

## 4. Discussion

Asthma is a dynamic disease in which pulmonary function, asthmatic symptoms, and physiological parameters fluctuate in general, and even more so in response to environmental perturbation, such as RV infection. We hypothesized that investigation of the temporal behavior of genetic regulation such as miRNAs in exosomes and inflammatory and immune biomarkers may unveil important underlying mechanisms hitherto unidentified in RV-induced asthma. In the current investigation, we compared RV-induced temporal responses of pulmonary function measurements in asthmatics and their healthy counterparts. Although routine longitudinal models did not reveal pulmonary function decline in asthmatics after the RV challenge, we did observe significant changes in ExoMiRNAs and cytokines through the analysis of their temporal dynamics. These findings indicate that although the low dose of the RV did not induce significant pulmonary function decline in our corticosteroid naïve mild asthmatic cohort, it triggered systemic post-transcriptional regulation and a distinct cytokine-mediated immune response. This is in line with our expectations with regard to the dynamics of the changes occurring at the molecular level being faster than the changes reflected at the clinical level measured via the lung function measurements. In the current study, we present a novel investigation of the dynamics of ExoMiRNAs and their link with asthma symptoms in RV-induced asthma.

Exosomes are extracellular vesicles (30–150 nm) that carry macromolecules, including miRNAs, thereby enabling intercellular communication. Recently, an increasing number of studies have shown the involvement of circulating exosomal miRNAs in mediating inflammatory processes in respiratory diseases, such as asthma [15,41,42]. Due to the availability and stability of exosomal miRNAs, they have become promising minimally invasive biomarkers [43,44]. MiRNAs have also been shown to mediate immune responses in respiratory virus infections, such as respiratory syncytial virus (RSV) [16,45] and RV [45]. Despite the implications of the regulatory roles of miRNAs in asthma and respiratory infections, there is little information to date about the roles of exosomal miRNAs in RV-induced asthma.

We performed a longitudinal analysis of ExoMiRNAs expression and found that a greater number of miRNAs were differentially expressed in the asthmatic subjects compared to healthy subjects between before and after the RV challenge. We also showed that before the RV challenge, miRNA expression in asthmatics did not differ from those in healthy subjects. In comparison, following the RV challenge, 26 miRNAs were found to be differentially expressed in asthmatics compared to healthy subjects. Notably, these 26 miRNAs were also differentially expressed between pre- and post-RV challenge phases in asthmatics, indicating a central role in regulating cellular functions under viral infection in asthma. We identified two distinct clusters within the DE miRNAs with different dynamics following the RV challenge, where the mean expression of the Upregulated Cluster was upregulated in asthmatic subjects compared to healthy subjects after the initial downregulation, and the Downregulated Cluster miRNAs were downregulated in asthmatic subjects after the initial upregulation. We show that the different dynamics of DE miRNAs were correlated with distinct immune responses, pointing towards potentially different mechanisms.

Upregulated Cluster miRNAs were significantly correlated with Th1 and interferon-induced cytokine/chemokine (IFN-γ and IP-10). RV16 induces IFN-γ and IP-10 [46,47], and deficient IFN-γ responses to antigenic triggers such as RV have been implicated in asthma and may be associated with asthma severity [5]. Consistent with these findings, we found that IFN-γ and IP-10 were induced by RV challenge, and more importantly, IFN-γ response to rhinovirus was dampened in asthmatic subjects compared to their healthy counterparts. In addition to its anti-viral function [48,49], IFN-γ inhibits Th2 cell differentiation and impaired IFN-γ promotes susceptibility to infection in asthmatics [50,51]. Thus, the strong correlation between Upregulated Cluster and Th1 suggests the critical roles of these miRNAs in anti-viral and anti-inflammatory functions. Additionally, the Upregulated Cluster was strongly correlated with Reg (IL-10). IL-10 has immunoregulatory functions and its level is inversely correlated with rhinoviral load [52,53]. A prior study showed that impaired Th1/IL-10 responses were associated with increased RV-induced symptoms [53]. We noted that IL-10 was induced by RV but was not differentially induced in asthma, suggesting the anti-viral immunity is accompanied by the regulatory cytokine IL-10 in both healthy and asthmatic subjects. Taken together, Upregulated Cluster miRNAs may play important roles in anti-viral immunity, and our subsequent gene enrichment analysis of Upregulated Cluster miRNAs supports their roles in anti-viral defensive mechanisms.

Downregulated Cluster miRNAs were strongly correlated with Th2 (IL-13), indicating their potential roles in Th2 immunity. Gene enrichment analysis of Downregulated Cluster miRNAs, however, suggests that these miRNAs mostly mediate pathogen recognition (positive regulation of Toll-like receptor (TLR) 7 signaling pathway and TLR9 signaling pathway) and anti-viral defensive mechanisms. The lack of correlation between Downregulated Cluster miRNAs and IFN itself could be explained by the indirect association between Downregulated Cluster miRNAs and Th1 immunity via the crosstalk between Th1 and Th2 cells [54]. For example, Th2 cytokines such as IL-13 and IL-4 have been found to impair innate immune responses, such as IFN productions against RV infection [55,56]. Strikingly, RV suppressed IL-17A secretion in healthy subjects. IL-17A contributes to the recruitment of neutrophils and modulates epithelial responses to RV [57]. We speculate that the suppression of IL-17A in healthy subjects may protect them from inducing a neutrophilic inflammatory response in the airways.

In line with previous studies [58,59], our results show that RV induces the secretion of IL-6 in healthy subjects. IL-6 is a proinflammatory but pleiotropic cytokine, and has been recently found to be essential for suppression of inflammation caused by viruses [60]. The robust IL-6 response in healthy subjects may protect them from inflammation. Interestingly, while neither the Upregulated Cluster nor the Downregulated Cluster miRNAs are correlated with proinflammatory cytokines (Inflam: IL-1β, IL-6, IL-8, and TNF-α), the pooled miRNAs are correlated with Inflam, indicating that their interactions between the miRNAs in both clusters promote proinflammatory processes. Further investigations are needed to examine the proinflammatory roles of these miRNAs in RV-induced asthma. In contrast to earlier reports in primary bronchial epithelial cells [61] and airway mucosal lining fluid [62], IL-33 induction was absent in the nasal lavage from either of the subject group. IL-33 is an epithelial cell-derived cytokine, and the different location could explain the discrepancy between our results and the others.

Allergic asthmatics are characterized by type 2 inflammation in the airways and thus it is not surprising to observe a significantly higher level of FeNO and eosinophil percentage in the asthmatics at baseline. Interestingly, the RV challenge failed to perturb the FeNO and eosinophil percentage levels in these subjects. Lewis et al. previously showed that an elevated FeNO level depended on the virus infection status and that only subjects with virus-negative cold-like illness demonstrated an increase [63], and this observation could explain the lack of perturbation of FeNO in our cohort as they already had higher baseline FeNO levels. Despite the lack of eosinophilic response to the rhinovirus challenge, we observed an increased level of neutrophil percentage in asthmatic subjects. Neutrophilic infiltration is implicated in RV-induced diseases [64,65]. We observed the concurrent induction of IL-8 (a potent chemoattractant for neutrophil [66]) and neutrophil percentages from nasal lavage in our study, indicating that neutrophils may be recruited to the airway through the mediation of IL-8. The high correlation between Downregulated Cluster miRNAs and FeNO suggests that Downregulated Cluster miRNAs can reflect airway inflammation, but the lack of association with eosinophil percentage or neutrophil percentage may indicate that Downregulated Cluster miRNAs do not regulate airway inflammation via eosinophils or neutrophils.

To dissect the functions of the miRNAs in the Upregulated Cluster and Downregulated Cluster, we performed gene enrichment analysis and proposed the potential miRNA–target gene regulatory network. Notably, although there was no overlap between the miRNAs in the Upregulated and Downregulated Cluster, the miRNAs in the two clusters target 113 common genes and 23 common GO terms. Not surprisingly, given the strong correlations between Upregulated Cluster miRNAs and IFN, we identified genes and enriched pathways contributing to anti-viral immunity. Furthermore, Upregulated Cluster miRNAs mediate MAPK cascade (Table S9). Previously, RV16 was found to activate the MAPK pathway, and inhibition of the MAPK pathway significantly increased RV16-induced IP-10 [67]. Enrichment analysis in genes targeted by Downregulated Cluster miRNAs revealed defense responses through positive regulation of TLR7 and TLR9 signaling pathways. TLR7 deficiency leads to impaired innate immune responses to RV in asthma, and blocking hsa-miR-150, hsa-miR-152, and hsa-miR-375 was shown to restore TLR7 and IFN responses [68]. Similarly, we observed an increased level of hsa-miR-375 one day after the RV challenge, but then observed decreasing trend of hsa-miR-375 expression. Interestingly, hsa-miR-375 (Downregulated Cluster) was highly correlated with Th13, Th17, pulmonary function, and inflammatory biomarkers (canonical correlation: 0.2189, −0.1827, −0.2271, and 0.2139, respectively; data not shown). Despite the absence of hsa-miR-375 in TargetScan and Tarbase, it may play crucial roles in regulating the TLR7 signaling pathway along with other miRNAs in the Downregulated Cluster. A TLR9 agonist shows the capacity to restore the Th2/Th1 imbalance in asthma by inducing type 1 responses [69]. The identification of “positive regulation of TLR9” by Downregulated Cluster miRNAs indicates that the process of restoring Th2/Th1 imbalance in asthma may be accompanied by miRNA regulation. In the common GO term “Viral process”, Downregulated Cluster miRNA hsa-miR-101-3p targets IFIH1 (interferon induced with helicase C domain 1), whose product MDA5 specifically senses rhinoviruses [70]. Upregulated Cluster miRNAs hsa-miR-21-5p and hsa-miR-126-3p and Downregulated Cluster miRNA hsa-miR-4433b-3p target IFN-stimulated gene RSAD2 (also known as viperin), a gene playing important roles in anti-viral and host defensive responses in RV infection [71].

Our study focused on treatment naïve allergic asthmatics in order to assess the dynamics of an unperturbed immune response to RV infection and is unique in its longitudinal design. In the era of personalized medicine, our results help us understand the mechanisms underlying different phenotypes of asthma. For example, IFN-stimulated genes and pathways were identified in the comparison of our allergic asthma versus controls, as well as in the obesity-associated low type-2 asthma cohort; however, in contrast to obesity-associated asthma, we did not identify gap junction pathways or its related genes [72]. In addition to the limitations previously discussed by Sinha et al. [18], we acknowledge that using an independent cohort with mild to moderate asthmatics who were on inhaled corticosteroids to guide the identification of target genes may not accurately identify genes involved in the treatment naïve asthma, but it is still likely to be representative given that the two cohorts were inoculated with identical doses of RV16. Due to the uniqueness of our study design, it was the most similar publicly available dataset. In our current investigation, we present potential gene targets that are regulated by the DE ExoMiRNAs; however, our results merit validation with a larger cohort. We also acknowledge that pulmonary function, inflammatory biomarkers, and cytokine responses may manifest perturbations by RV at different times. We attempted to investigate the correlations between DE ExoMiRNAs and these three different compartments with varying time lags, but we were limited by the relatively low sampling frequency of serum compared to the other clinical features.

## 5. Conclusions

In conclusion, we uniquely show that ExoMiRNAs are perturbed by the nasal RV challenge, and their expression is altered in RV-induced asthma. Time series analysis of miRNAs identifies different clusters that are correlated with different cytokines, inflammatory biomarkers, and pulmonary function. Our results suggest that despite the different miRNA–trait correlations and enriched gene pathways, the two miRNA clusters target key genes that both play critical roles in the anti-viral process. Our findings provide insights into the regulatory roles of ExoMiRNAs and their link with asthma features in RV-induced asthma may serve as biomarkers and provide novel targets for preventing asthma exacerbations.

## Figures and Tables

**Table 1:** Characteristics of healthy and asthmatic subjects at baseline (modified from [18]). The *p*-value was calculated by *t*-test for continuous variable or Fisher’s exact test for categorical variable.

Demographic Features	Healthy (n = 12)	Asthmatic (n = 12)	*p*-Value
Age (years), mean ± sd	21 ± 1.5	22.2 ± 2.2	0.181
Female gender, n (%)	7 (58.3%)	8 (66.7%)	1
Ethnicity (Caucasian), n	11	9	0.590
BMI, mean ± sd	22.2 ± 1.6	22.8 ± 3.1	0.576
Smoking (pack years), n	1 (0.17 PY)	NA	NA
Height (cm), mean ± sd	177.7 ± 8.6	172.5 ± 13.0	0.260
Weight (kg), mean ± sd	70.4 ± 10.1	67.8 ± 12.4	0.575
Baseline FEV1% predicted, mean ± sd	105.7 ± 11.6	101.0 ± 10.0	0.507
Baseline FVC% predicted, mean ± sd	104.2 ± 10.5	104.2 ± 10.2	0.931
Baseline PEF% predicted, mean ± sd	108.4 ± 14.0	104.7 ± 12.2	0.624

**Table 2:** Mixed-effects regression model results for testing pulmonary function, inflammatory biomarkers, and cytokine responses between asthmatic and healthy subjects. Coefficient estimates (β), standard error (SE), and *p*-value were reported for Time (${}^{‡}$), Group (${}^{ℵ}$), and Time-by-Group interaction ((Time × Group); ${}^{†}$). All responses were log-transformed to promote the normality of residuals. Time × Group indicates whether RV challenge perturbed the measuring response differently between asthmatic and healthy subjects. Time indicates whether the RV challenge induced significant changes in the measuring response in healthy subjects. Group indicates whether the corresponding measurement was different between asthmatic and healthy subjects at baseline.

		Time × Group ^†^		Time ^‡^		Group ^ℵ^	
		β (SE)	*p*-Value	β (SE)	*p*-Value	β (SE)	*p*-Value
**Pulmonary function**	FVC%	$5.820\times 10^{-3}$ ($5.912\times 10^{-3}$)	0.3269	−0.0111 ($4.173\times 10^{-3}$)	0.0146	0.0202 (0.05698)	0.723
	FEV${}_{1}$%	$3.61\times 10^{-4}$ ($4.812\times 10^{-3}$)	0.9403	$-5.40\times 10^{-3}$ ($3.397\times 10^{-3}$)	0.126	$-3.4\times 10^{-4}$ (0.04953)	0.995
	PEF%	−0.0114 ($8.243\times 10^{-3}$)	0.1683	$-3.07\times 10^{-3}$ ($5.828\times 10^{-3}$)	0.599	−0.0294 (0.09050)	0.745
**Inflammatory biomarkers**	FeNO	−0.0159 (0.0303)	0.606	$-5.26\times 10^{-6}$ (0.0214)	0.999	1.072 (0.143)	<0.0001
	Eosinophil%	−0.465 (0.255)	0.0706	0.0853 (0.172)	0.621	6.193 (1.454)	0.0003
	Neutrophil%	0.471 (0.220)	0.0435	−0.379 (0.148)	0.0182	−0.619 (0.282)	0.0399
**Cytokine**	IFN-γ ^§^	0.139 (0.0455)	0.0057	−0.0643 (0.0298)	0.0424	0.311 (0.485)	0.527
	IP-10	−0.0902 (0.0482)	0.0744	0.0784 (0.0310)	0.0191	−0.142 (0.266)	0.598
	IL-13	−0.0122 (0.0199)	0.548	0.0825 (0.01289)	<0.0001	−0.126 (0.110)	0.265
	IL-17A ^§^	−0.112 (0.0355)	0.0047	0.140 (0.0226)	<0.0001	−1.239 (0.358)	0.0022
	IL-10	0.101 (0.0518)	0.0644	0.209 (0.0324)	<0.0001	−0.748 (0.205)	0.0014
	IL-1β ^§^	−0.0700 (0.0348)	0.0562	0.102 (0.0220)	0.0001	−0.966 (0.362)	0.0141
	IL-6	−0.272 (0.0602)	0.0002	0.301 (0.0386)	<0.0001	0.584 (0.328)	0.0884
	IL-8 ^§^	−0.0544 (0.0283)	0.0680	0.0409 (0.0188)	0.0401	−1.118 (0.322)	0.0806
	TNF-α	−0.0196 (0.0339)	0.569	0.151 (0.0212)	<0.0001	0.0270 (0.171)	0.876
	IL-33	−0.00681 (0.0622)	0.913	−0.0219 (0.0412)	0.597	−0.0510 (0.214)	0.814

^§^ Quadratic time term was used.

**Table 3:** DE miRNA cluster membership identified by time series clustering analysis. Two clusters of miRNAs were identified from DE miRNAs using the log2 fold change in asthmatic subjects relative to healthy subjects post-RV challenge.

Upregulated Cluster	Downregulated Cluster
hsa-let-7f-5p	hsa-miR-122-5p
hsa-miR-26a-5p	hsa-miR-148a-3p
hsa-let-7a-5p	hsa-miR-101-3p
hsa-miR-92a-3p	hsa-miR-151a-3p
hsa-miR-21-5p	hsa-miR-375-3p
hsa-miR-126-3p	hsa-miR-10b-5p
hsa-miR-30c-5p	hsa-miR-423-5p
hsa-miR-22-3p	hsa-miR-671-3p
hsa-miR-199a-3p	hsa-miR-127-3p
hsa-miR-221-5p	hsa-miR-4433b-3p
hsa-miR-103a-3p	hsa-miR-99a-5p
hsa-miR-1-3p	

**Table 4:** Top 15 enriched gene ontology (GO) terms in each DE miRNA cluster.

	GO Term	Fisher Exact *p*-Value
**Upregulated Cluster**	defense response to virus	$8.10\times 10^{-6}$
	negative regulation of viral genome replication	$4.20\times 10^{-6}$
	response to virus	$1.20\times 10^{-5}$
	type I interferon signaling pathway	$4.50\times 10^{-5}$
	epidermal growth factor receptor signaling pathway	$1.00\times 10^{-4}$
	positive regulation of phosphatidylinositol 3-kinase signaling	$2.50\times 10^{-4}$
	protein phosphorylation	$9.60\times 10^{-4}$
	RNA phosphodiester bond hydrolysis, endonucleolytic	$4.70\times 10^{-4}$
	response to glucose	$6.50\times 10^{-4}$
	glycosaminoglycan metabolic process	$2.10\times 10^{-4}$
	heparan sulfate proteoglycan biosynthetic process	$2.70\times 10^{-4}$
	macromolecular complex assembly	$1.50\times 10^{-3}$
	viral process	$5.10\times 10^{-3}$
	protein polyubiquitination	$5.00\times 10^{-3}$
	cellular response to insulin stimulus	$3.60\times 10^{-3}$
**Downregulated Cluster**	negative regulation of viral genome replication	$2.20\times 10^{-8}$
	response to virus	$3.00\times 10^{-6}$
	type I interferon signaling pathway	$8.10\times 10^{-5}$
	viral process	$1.40\times 10^{-3}$
	protein polyubiquitination	$2.70\times 10^{-3}$
	defense response	$1.40\times 10^{-3}$
	positive regulation of interferon-beta production	$9.30\times 10^{-4}$
	phospholipid transport	$1.00\times 10^{-3}$
	positive regulation of protein export from nucleus	$1.10\times 10^{-3}$
	brain development	$5.80\times 10^{-3}$
	proteasome-mediated ubiquitin-dependent protein catabolic process	$5.10\times 10^{-3}$
	cellular response to virus	$2.50\times 10^{-3}$
	negative regulation of apoptotic process	$1.00\times 10^{-2}$
	negative regulation of viral entry into host cell	$1.70\times 10^{-3}$

## Data Availability

All data used in this study are available on request from the corresponding author.

## Supplementary Materials

The following supporting information can be downloaded at: https://www.mdpi.com/article/10.3390/v14112444/s1, Figure S1. Pulmonary function in response to RV challenge in asthmatic (magenta) and healthy subjects (blue). Figure S2. Exosome characterization. Figure S3. Representative trends of differentially expressed miRNAs before and after RV challenge. Figure S4. MiRNA–target gene regulatory networks for the Upregulated Cluster and the Downregulated Cluster. Table S1. DE miRNAs between asthmatic and healthy subjects after RV challenge (between-challenged). Table S2. DE miRNAs pre- versus post-RV challenge within healthy subjects (within-healthy). Table S3. DE miRNAs pre- versus post-RV challenge within asthmatic subjects (within-asthmatic). Table S4. Correlations between the clinical traits and the first canonical variate of DE miRNAs, Upregulated Cluster, and Downregulated Cluster. Table S5. The DE miRNAs and their target genes. Table S6. The Upregulated Cluster miRNAs and their target genes. Table S7. The Downregulated Cluster miRNAs and their target genes. Table S8. GO terms enriched by the DE miRNAs. Table S9. GO terms enriched by the Upregulated Cluster miRNAs. Table S10. GO terms enriched by the Downregulated Cluster miRNAs.

## Abbreviations

The following abbreviations are used in this manuscript:

miRNA	microRNA
RV	rhinovirus
RSV	respiratory syncytial virus
FeNO	fractional exhaled nitric oxide
IFN-γ	interferon-gamma
IL	interleukin
IP-10	IFN-γ-inducible protein-10
DE	differentially expressed
TNF-α	tumor necrosis factor alpha
FVC	forced vital capacity
FEV${}_{1}$	forced expiratory volume in the first second
PEF	peak expiratory flow
ExoMiRNAs	exosomal microRNAs
exoRNAs	exosomal RNAs
Th1	T-helper cell type 1
Th2	T-helper cell type 2
PACE	principal components analysis through conditional expectation
DEGs	differential expressed genes
