# Supplementary material for: Analysis of Exosomal MicroRNA Dynamics in Response to Rhinovirus Challenge in a Longitudinal Case-Control Study of Asthma
Source: Viruses. 2022 Nov 3;14(11):2444. doi: 10.3390/v14112444 (PMC9695046; doi:10.3390/v14112444)
Supplement: Supplementary file 1 [file viruses-14-02444-s001.zip › Supplementary_material.pdf]

Analysis of Exosomal MicroRNA Dynamics in Response to Rhinovirus Challenge in a Longitudinal Case-Control Study of Asthma

Wangfei Wang, Anirban Sinha, René Lutter, Jie Yang, Christian Ascoli, Peter J. Sterk, Nicole K. Nemsick, David L. Perkins and Patricia W. Finn

SUPPLEMENTARY MATERIAL

Supplementary Methods

Characterization of exosomes

We selected three independent serum samples as laboratory controls to characterize exosomes. The size distribution and concentration of the nanoparticles were characterized using NanoSight NS300 (Malvern Panalytical) at the Research Resources Center of University of Illinois at Chicago. The exosome-containing pellet was resuspended in 150 μl PBS solution, and then further diluted for 150-fold before characterization. PBS solution was filtered through 0.02 μm filter before resuspending the pellets. Filtered PBS solution alone was used as a negative control.

Supplementary Table Captions

Supplementary tables **Table S1 – S3** are found on the file:

Supplementary_Tables_full_list_of_DE_miRNAs.xlsx

Supplementary table **Table S4** is found on the file: ***Supplementary_Tables_cca.xlsx***

Supplementary tables **Table S5 – S7** are found on the file:

Supplementary_Tables_target_genes.xlsx

Supplementary tables **Table S8 – S10** are found on the file:

Supplementary_Tables_enriched_GO_terms.xlsx

Supplementary Table S1 (Table S1): Differentially expressed (DE) miRNAs between asthmatic and healthy subjects after RV challenge (between-challenged).

Supplementary Table S2 (Table S2): DE miRNAs pre- versus post-RV challenge within healthy subjects (within-healthy).

Supplementary Table S3 (Table S3): DE miRNAs pre- versus post-RV challenge within asthmatic subjects (within-asthmatic).

Supplementary Table S4 (Table S4): Correlations between the clinical traits and the first canonical variate of DE miRNAs, Upregulated Cluster and Downregulated Cluster. Only the significant pairs are shown.

Supplementary Table S5 (Table S5): The DE miRNAs and their target genes

Supplementary Table S6 (Table S6): The Upregulated Cluster miRNAs and their target genes.

Supplementary Table S7 (Table S7): The Downregulated Cluster miRNAs and their target genes.

Supplementary Table S8 (Table S8): Gene Ontology (GO) terms enriched by the DE miRNAs.

Supplementary Table S9 (Table S9): GO terms enriched by the Upregulated Cluster miRNAs.

Supplementary Table S10 (Table S10): GO terms enriched by the Downregulated Cluster miRNAs.

Supplementary Figure S1 (Figure S1)

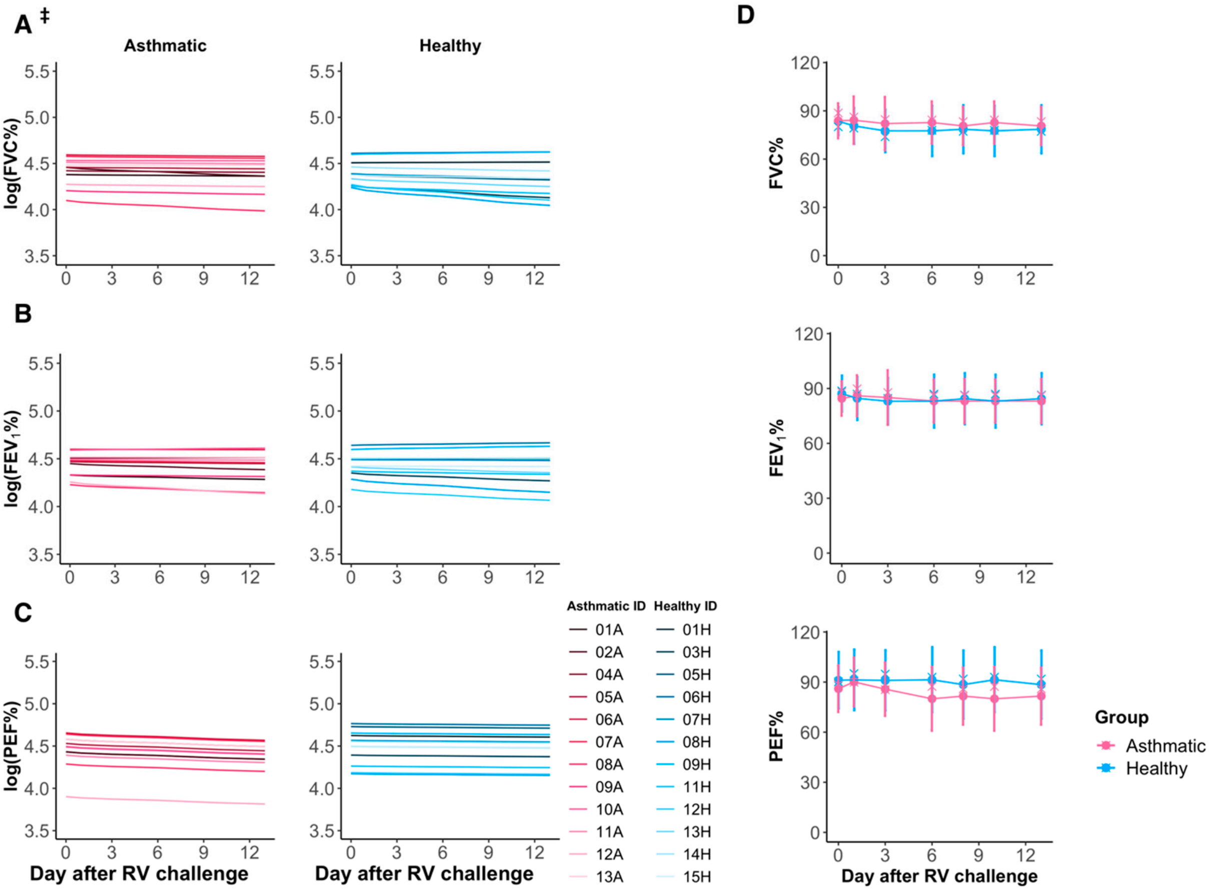

Figure S1. Pulmonary function in response to RV challenge in asthmatic (magenta) and healthy subjects (blue). Mixed-effects models for log-transformed levels of (**A**) FVC%, (**B**) FEV_1% and (**C**) PEF%. The pulmonary function measurements were plotted for each subject due to the high variability between subjects. Day 0 represents the averaged levels at baseline. ‡ represents the RV challenge induced the measuring response significantly in healthy subjects (Time variable in **Table 1**). (**D**) Mean (circle) and median ("x") of FVC%, FEV_1% and PEF% in asthmatic and healthy subjects after RV challenge. Error bars indicate standard deviation. No statistical difference was observed between asthmatic and healthy groups (Mann-Whitney U test; $p > 0.05$).

Supplementary Figure S2 (Figure S2)

A B

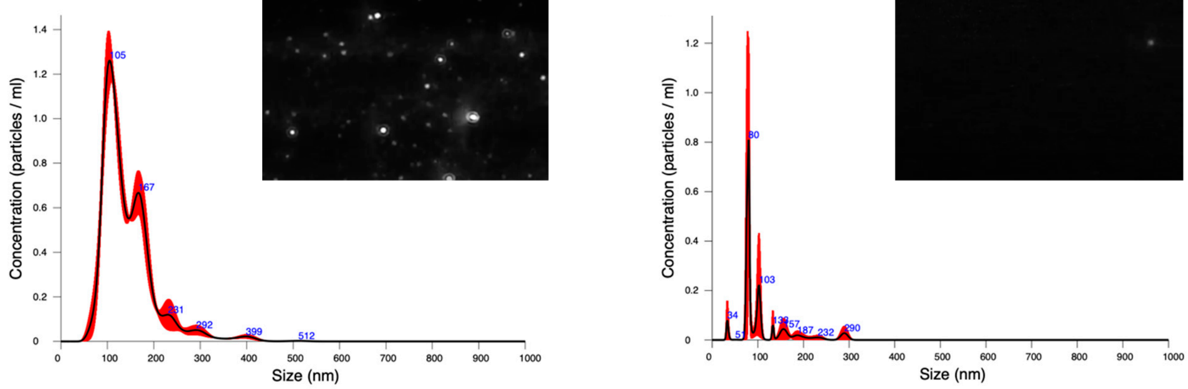

Figure S2. Exosome characterization. A laboratory control was utilized as proof of exosomal extraction. (**A**) Size distribution of serum-extracted exosomes. The size distributions from three samples were overlayed (red) and most of the nanoparticles had a diameter around 105 nm. (**B**) Size distribution of filtered PBS solution as negative control. Three PBS solution was used. Top right inserts are representative images from NanoSight.

Supplementary Figure S3 (Figure S3)

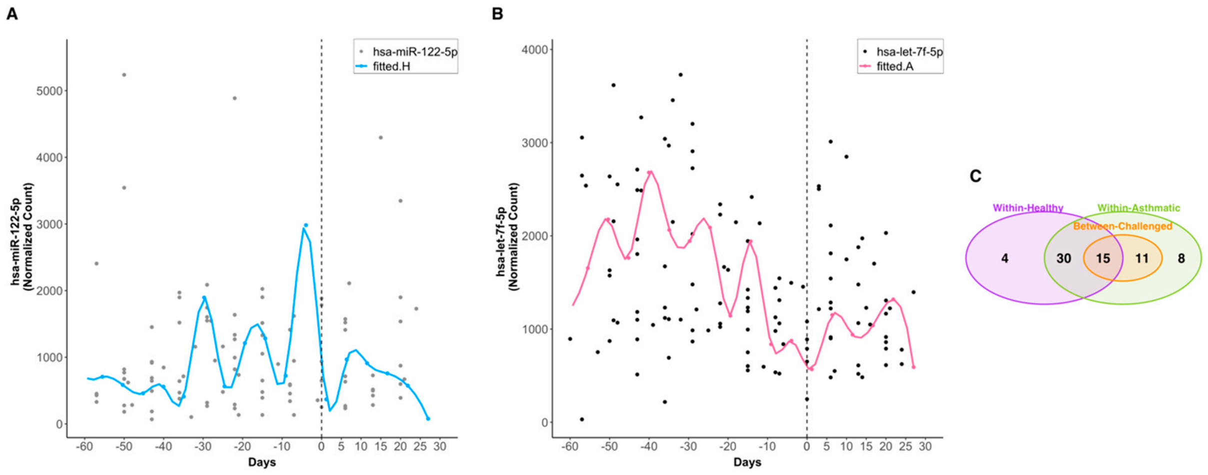

Figure S3. Representative trends of differentially expressed miRNAs before and after RV challenge. (**A**) Differential miRNA expression between pre- and post-RV challenge phases in healthy subjects (within-healthy). The blue line is the fitted smoothing spline, and the dots are the observed normalized hsa-miR-122-5p counts from 12 healthy subjects. (**B**) Differential miRNA expression between pre- and post-RV challenge phases in asthmatic subjects (within-asthmatic). The magenta line is the fitted smoothing spline, and the dots are the observed normalized hsa-let-7f-5p counts from 12 asthmatic subjects. (**C**) Venn diagram showing the number of overlapped miRNAs between each group.

Supplementary Figure S4 (Figure S4)

A

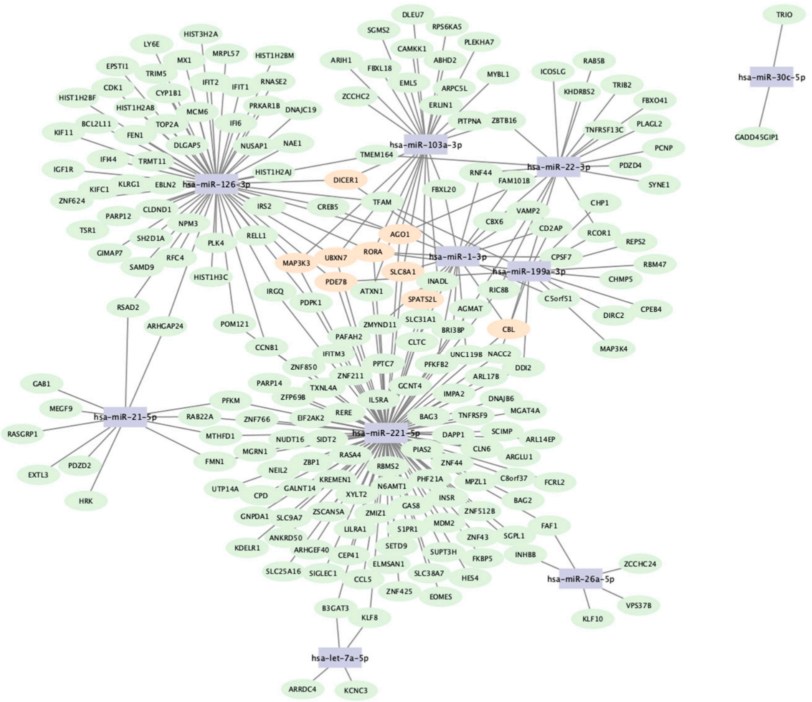

B

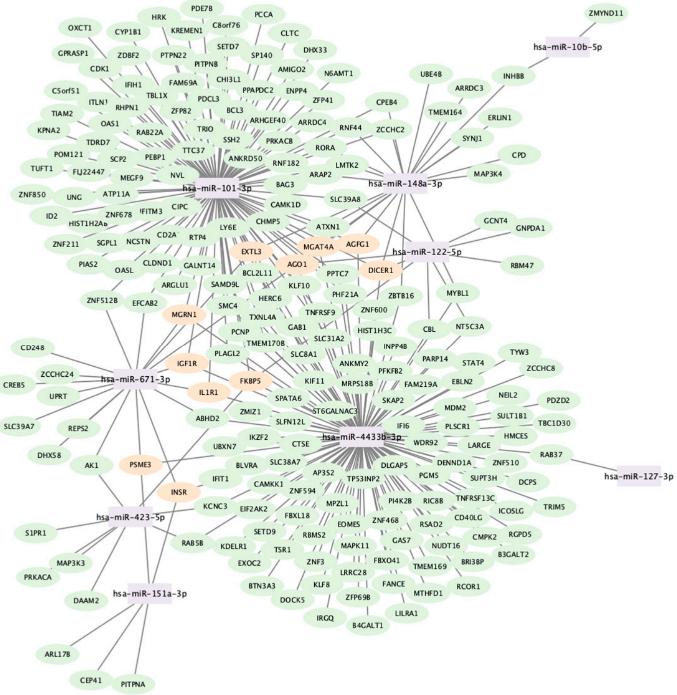

Figure S4. MiRNA-target gene regulatory networks for the (A) Upregulated Cluster and the (B) Downregulated Cluster. Purple rectangle boxes depict DE miRNAs. The green ellipses are predicted target genes, and the orange ellipses are the genes that were targeted by at least three miRNAs. Hsa-let-7f-5p and hsa-miR-92a-3p in Cluster 1 and hsa-miR-99a-5p in Cluster 2 were not shown on the networks because there was no overlapped gene between TargetScan/Tarbase predicted genes and the DEGs dataset.
