# Supplementary figures and images for: Analysis of Exosomal MicroRNA Dynamics in Response to Rhinovirus Challenge in a Longitudinal Case-Control Study of Asthma
Source: Viruses. 2022 Nov 3;14(11):2444. doi: 10.3390/v14112444 (PMC9695046; doi:10.3390/v14112444)

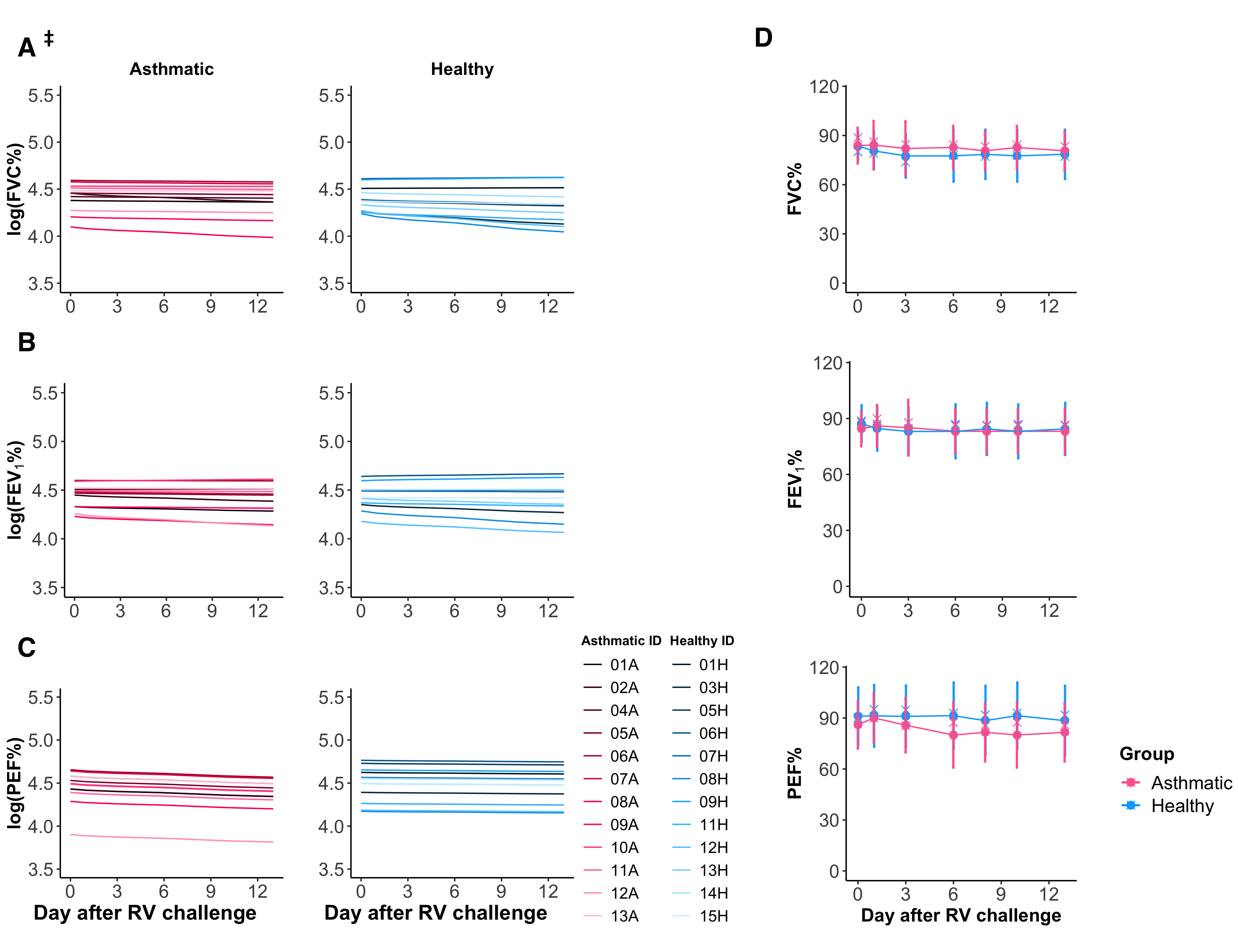

Supplement: Supplementary file 1 [file viruses-14-02444-s001.zip › Figure S1.png]

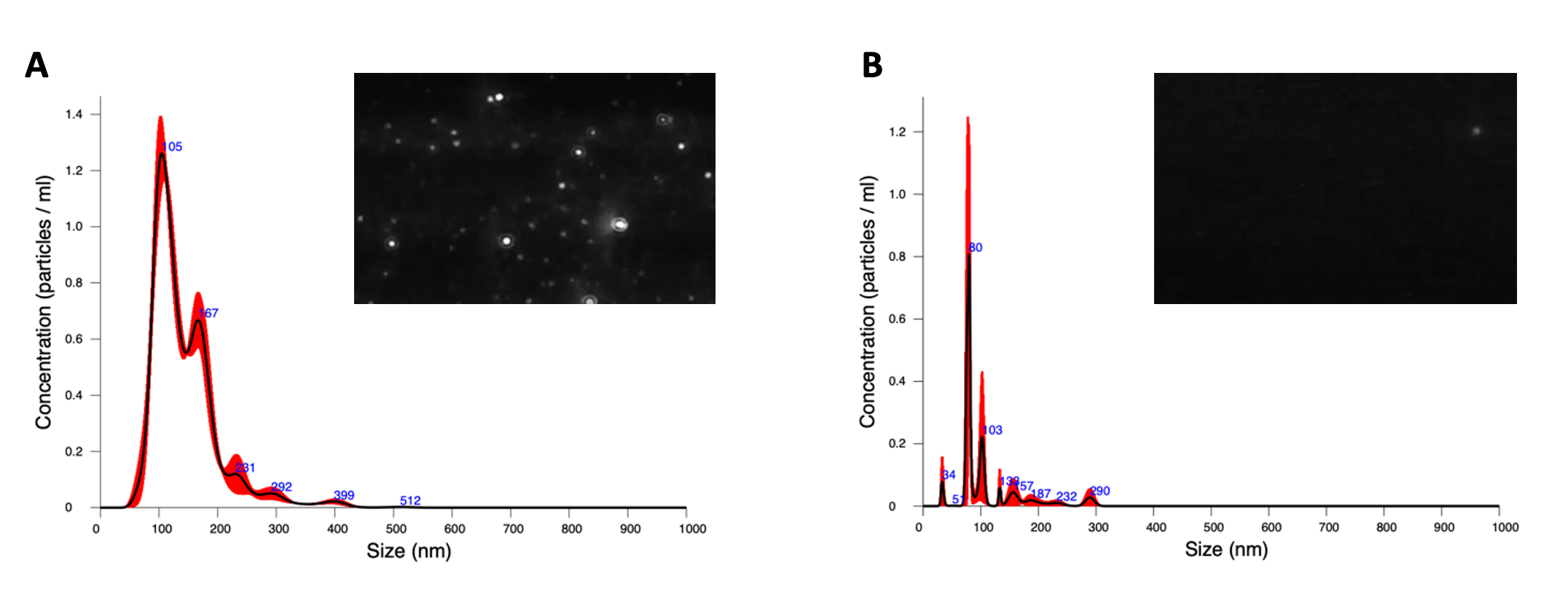

Supplement: Supplementary file 1 [file viruses-14-02444-s001.zip › Figure S2.png]

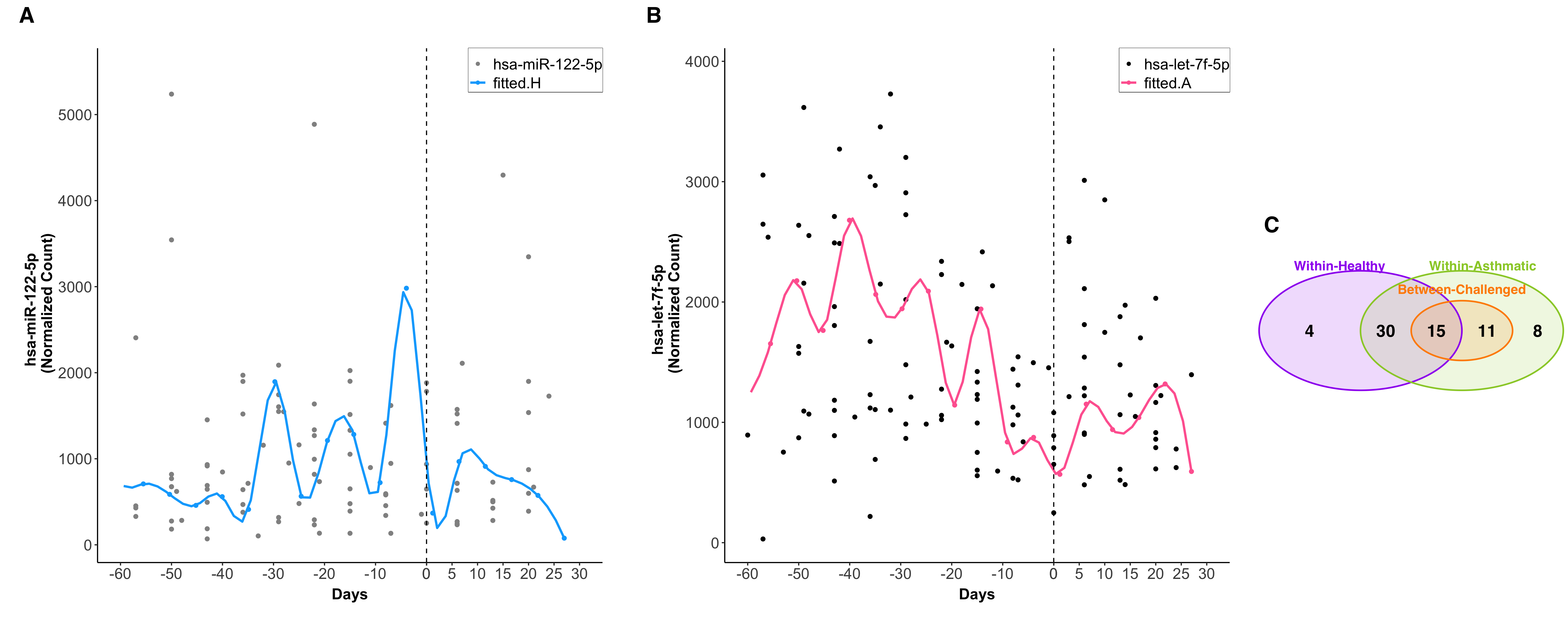

Supplement: Supplementary file 1 [file viruses-14-02444-s001.zip › Figure S3.png]

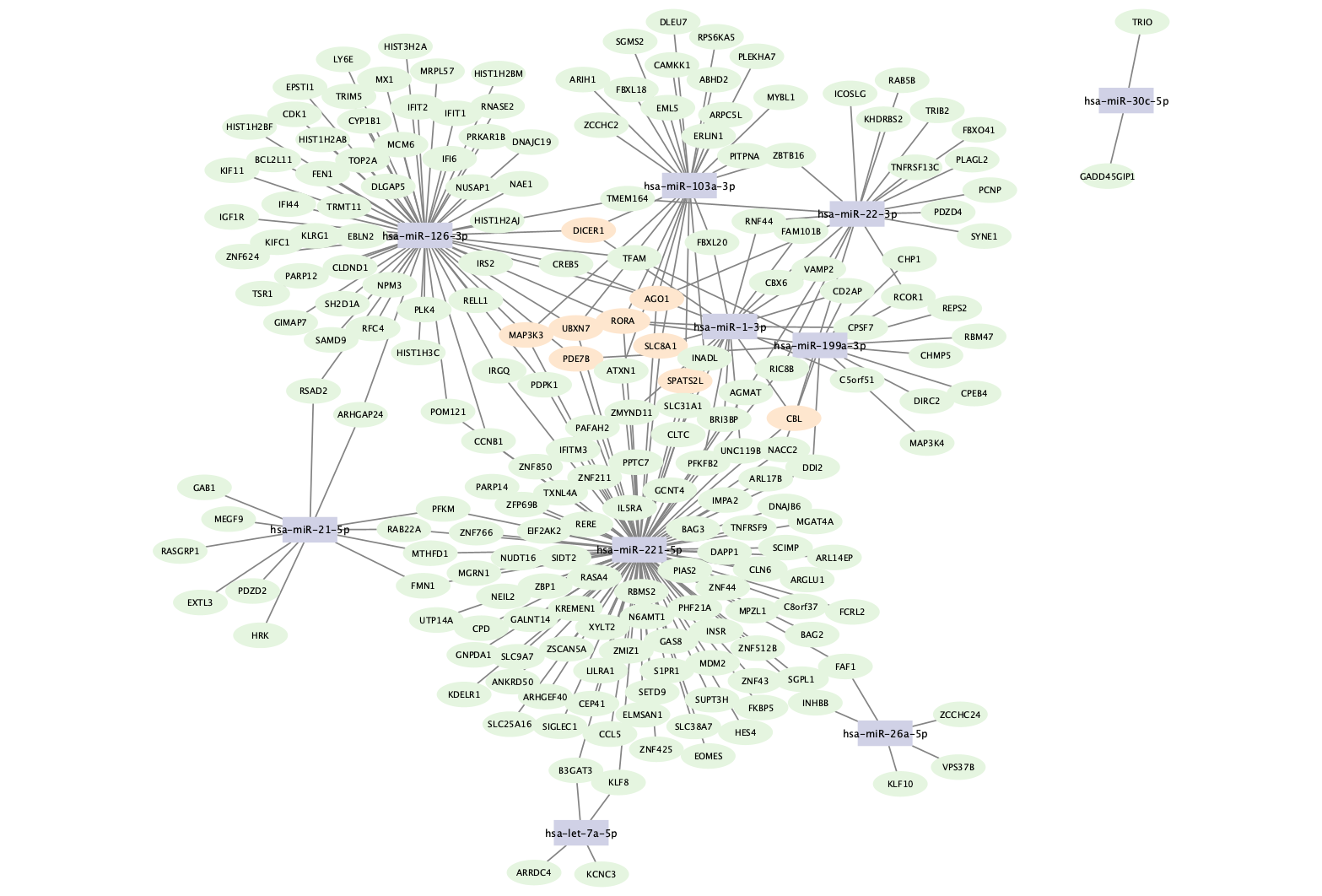

Supplement: Supplementary file 1 [file viruses-14-02444-s001.zip › Figure S4A. upclustergeneinref.txt.png]

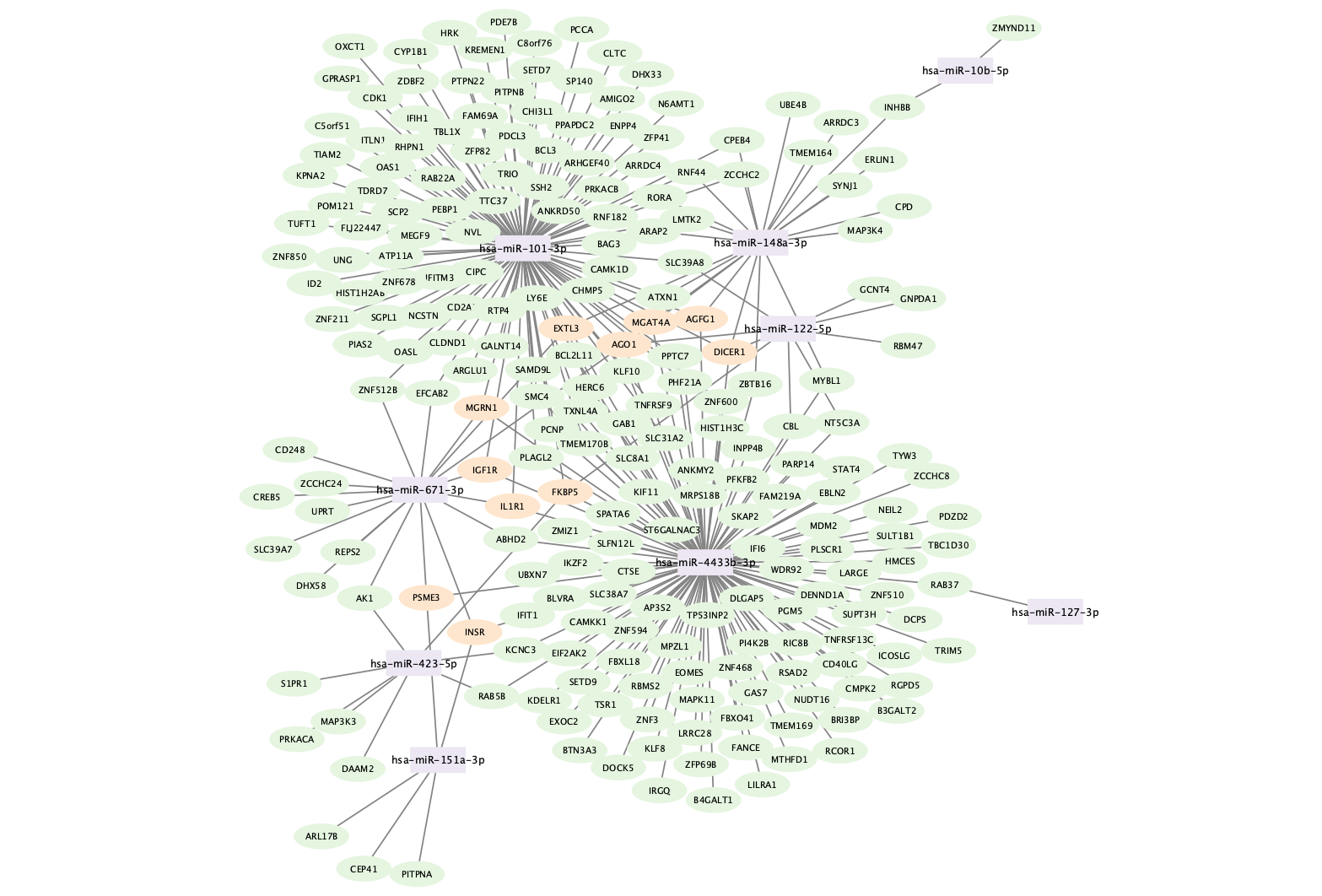

Supplement: Supplementary file 1 [file viruses-14-02444-s001.zip › Figure S4B. downclustergeneinref.txt.png]
